# Exercise Promotes Neuroplasticity in Both Healthy and Depressed Brains: An fMRI Pilot Study

**DOI:** 10.1155/2017/8305287

**Published:** 2017-07-30

**Authors:** Joanne Gourgouvelis, Paul Yielder, Bernadette Murphy

**Affiliations:** University of Ontario Institute of Technology, 2000 Simcoe Street North, Oshawa, ON, Canada L1H 7K4

## Abstract

Memory impairments are a frequently reported cognitive symptom in people suffering from major depressive disorder (MDD) and often persist despite antidepressant therapy. Neuroimaging studies have identified abnormal hippocampal activity during memory processes in MDD. Exercise as an ad-on treatment for MDD is a promising therapeutic strategy shown to improve mood, cognitive function, and neural structure and function. To advance our understanding of how exercise impacts neural function in MDD, we must also understand how exercise impacts healthy individuals without MDD. This pilot study used a subsequent memory paradigm to investigate the effects of an eight-week exercise intervention on hippocampal function in low-active healthy (*n* = 8) and low-active MDD (*n* = 8) individuals. Results showed a marked improvement in depression scores for the MDD group (*p* < 0.0001) and no change in memory performance for either group (*p* > 0.05). Functional imaging results showed a marginally significant decrease in hippocampal activity in both groups following the exercise intervention. Our whole brain analysis collapsed across groups revealed a similar deactivation pattern across several memory-associated regions. These results suggest that exercise may enhance neural efficiency in low-fit individuals while still resulting in a substantially greater mood effect for those suffering from MDD. This trial is registered with clinical trials.gov NCT03191994.

## 1. Introduction

Memory impairment is the most frequently reported cognitive symptom in people suffering from major depressive disorder (MDD) and often persists as a residual symptom following antidepressant therapy [1, 2]. Although the neural underpinnings of impaired memory in MDD remain unclear, research suggests that the hippocampus, which plays a critical role in the formation of new memories, also plays a role in the pathogenesis of MDD. To date, cognitive literature has presented mixed findings in terms of the type, severity, and specificity of memory deficits in people with MDD, although the plurality of data has suggested an impairment in episodic (autobiographical) memory with a sparing of semantic (general knowledge) memory and short-term memory [3–8]. The hippocampus has been shown to play an essential role in the encoding of episodic memories [5, 9–13], and pathologies associated with this neural structure may underlie the episodic memory impairments observed in MDD populations. The relationship between MDD, memory impairments, and hippocampal structure and function is based on converging lines of research from animal studies, neuroimaging, neuropsychology, and postmortem investigations which have all shown hippocampal abnormalities at the structural, functional, and cellular level. Structural brain imaging studies have shown robust hippocampal volume reductions particularly in persistent and early onset MDD [14–16]. Functional neuroimaging studies have found that both the memory encoding and retrieval processes within the hippocampus are to be impaired in MDD [4, 6, 17–19]. Neuropathological evidence from animal models of depression and postmortem studies in depressed humans have revealed cellular abnormalities in the hippocampus such as dendritic atrophy and reduced neuron and glial densities [20–24].

Despite many efforts to develop effective antidepressant therapies, MDD remains a severely undertreated disorder in the primary care setting leaving more than half of individuals plagued with symptoms [25–28]. Exercise as an add-on to conventional antidepressant therapies is a promising treatment strategy for MDD. It is well established that exercise is efficacious in treating mild to moderate depression with response rates comparable to mainstream therapies such as antidepressant medication and cognitive behavioral therapy [29–34]. However, there is a lack of understanding of the neurobiological mechanisms that underlie or mediate the antidepressant effects of exercise. It is well established that exercise facilitates neuroplasticity [35–37]. To date, much of our understanding of how exercise facilitates neural and cognitive plasticity has come from the extensive animal literature. For instance, rodent studies have shown that exercise increases new cells in the dentate gyrus of the hippocampus, and this is associated with improved learning and spatial memory [36, 38–40]. Further evidence has shown that exercise increases synaptogenesis [41] and angiogenesis [42] and improves dendritic morphology in the hippocampus [43, 44]. However, the effects of exercise on brain structure and function in humans have been more equivocal. In elderly populations, aerobics exercise training has been shown to improve spatial memory [45], executive function [46, 47], and short-term memory [48]; however, others observed no benefits [49–52]. Neuroimaging studies have found that aerobics exercise reverses age-associated brain volume loss in the prefrontal and temporal cortices [53, 54], improves functional connectivity in the default mode and frontal executive networks [47], and increases hippocampal volume in schizophrenics [55]. To date, the literature examining the effects of chronic exercise on neural function in both healthy and MDD populations remains scant. To capitalize on the full treatment potential of exercise in MDD populations, we must also understand the relationship between cardiovascular fitness, neural function, and cognitive performance in healthy individuals in order to identify neural mechanisms specific to MDD.

To our knowledge, this is the first study using a subsequent memory paradigm to determine the effects of an eight-week exercise prescription on the functional integrity of the hippocampus in low-active patients with MDD and low-active healthy individuals. The aim of this pilot study was twofold: (1) using fMRI to examine changes in hippocampal function following an exercise intervention, and (2) to conduct an exploratory whole brain analysis to determine how exercise affects overall brain activity.

## 2. Materials and Methods

### 2.1. Participants

Eight patients (mean age = 37.25, SD = 8.00; 7 females) with comorbid MDD and anxiety were recruited from an outpatient Mental Health Day Treatment Program at a local hospital in Oshawa, Ontario Canada. Eight healthy participants (mean age = 20.63, SD = 1.19; 4 females) with no history of mental health illness or neurological disease were recruited from a local university in Oshawa, Ontario Canada. Depressed patients had a diagnosis of MDD according to an unstructured clinical interview by hospital psychiatrists based on Diagnostic and Statistical Manual of Mental Disorders—Fourth Edition (DSM-IV-TR) [56] criteria, no coexisting DSM-V Axis I disorders apart from anxiety, and a score ≥20 on the Beck Depression Inventory—Second Edition (BDI-II) [57], and their pharmacological treatment was stabilized a minimum of six weeks prior to study enrolment. In order to be considered eligible for the study, participants needed to indicate that they exercised less than 20 minutes, three times per week. Both groups were also safety screened for MRI and screened with the Physical Activity Readiness Questionnaire (PAR-Q) to ensure they had no medical contraindications to exercise. All participants provided written consent.

### 2.2. Psychometric Evaluation

Participants completed the Montreal Cognitive Assessment (MoCA) which is a brief neurocognitive tool with high sensitivity for screening patients with mild cognitive impairment. This cognitive assessment was performed to identify participants who may have difficulty performing the associative memory task. The internal consistency of the MoCA is good, with a coefficient alpha of 0.83 [58]. Depression was measured using the Beck Depression Inventory (BDI-II) [57] which is one of the most widely used self-reported instrument capable of measuring depression severity ranging from not depressed to severely depressed. The BDI–II demonstrated excellent internal consistency, with a coefficient alpha of 0.91 [59]. All measures were performed before and after the eight-week exercise intervention.

### 2.3. Fitness Assessment

Cardiorespiratory fitness was measured before and after the exercise intervention using the YMCA cycle ergometer protocol recommended by the American College of Sports Medicine [60–62]. The YMCA cycle ergometer protocol is an indirect submaximal exercise test used to estimate maximal oxygen consumption (VO_2_max) from heart rate (HR) measurements. The protocol consists of two or more consecutive 3-minute stages at a given workload. The objective was to elevate the participant's HR to a target zone to approximately 85% of the age-predicted maximum HR for two consecutive stages. The initial workload consisted of a 25-watt workload at a cadence of 50 revolutions per minute. HR was measured and recorded using the radial pulse method during the final 15 seconds of each minute, which determined the workload of subsequent stages indicated by the YMCA protocol. Once a steady state HR (two successive measures that differ from <5 bpm) was within 10 bpm of the 85% age-predicted maximum HR, the test was complete. VO_2_max was estimated using an equation that includes workload, body mass, and derived constants.

### 2.4. Exercise Intervention

Participants performed an individualized eight-week exercise program consisting of three weekly sessions (described below). Exercise sessions were performed alone, on nonconsecutive days, and each session was supervised by a qualified exercise professional to increase compliance [34]. Attendance was recorded and all participants completed >80% of the exercise sessions.

The exercise prescription was based on international recommendation to obtain at least 150 minutes per week of moderate to vigorous intensity aerobics exercise and to perform strengthening activities twice per week, for developing and maintaining cardiorespiratory, musculoskeletal, and neuromotor fitness in healthy adults [63, 64]. This minimum-effective dose of exercise was prescribed to encourage better compliance since people with depression, and are low active, tend to be less motivated [65]. Research has also shown that combining aerobics with strength training improves depression and cognitive function such as attention, processing speed, executive function, and memory performance more than aerobics exercise alone [51, 66].

#### 2.4.1. Resistance Sessions

Resistance sessions were completed twice per week and incorporated a whole-body exercise prescription using larger muscle groups. Each session included eight resistance exercises using both resistance training machines and free weights. Initial workloads were approximately 95% of the 10 repetition maximum to ensure proper form. Exercises were performed in two or three supersets (one set of each exercise with no rest between sets) with an 8–12 repetition range in order to decrease rest times and to maintain target HR. Workload was increased approximately 5% once participants were able to complete three sets of 12 repetitions with proper form. Specific exercises were changed every four weeks; however, they targeted the same muscle groups. Resistance exercises included variations of the chest press, pull downs, triceps extension, biceps curl, shoulder press, leg press, leg extensions, leg curls, squats, split squats, calf raises, and abdominal exercises. During each session, HR was monitored to ensure that the participant maintained a HR of between 60–80% of their age-predicted maximum HR. Each session began with a 5-minute aerobics warmup and ended with 15 minutes of aerobics activity that was performed on either the treadmill, stationary bike, or elliptical trainer.

#### 2.4.2. Aerobics Session

Participants completed an aerobics-only session once per week. They were given the choice to perform their aerobics activity on either the treadmill, stationary bike, or elliptical trainer. The aerobics session workloads were determined by HR response and increased by five-minute increments over the eight weeks reaching a maximum of 60 minutes per session. HR was monitored throughout the session to ensure the participant was in the target HR range.

### 2.5. Statistical Analysis

Statistical analyses were performed using GraphPad Prism software, version 6.0 data. Data are presented as mean (standard deviation (SD)). *P* values less than 0.05 were considered significant. Differences in baseline variables between groups were tested using a two-tailed Student's *t*-test and chi-square test for gender distribution. Within group differences for pre-post-BDI, MoCA, BMI, and VO_2_max were tested using a paired *t*-test. A two-way repeated measures analysis of variance (ANOVA) was used to determine any group × time of interactions and to compare the changes between the two treatment groups. Cohen's *d* was used to represent the effect size within each group. For between group effect sizes, we used *d*_ppc2_ [67] which uses the difference between Hedge's *g* of two different treatment groups in pre-post research designs.

### 2.6. Associative Memory Task

**Figure 1:**
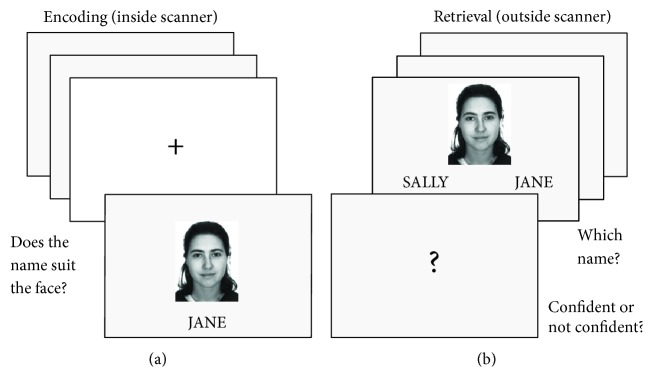
(a) During the encoding task, participants viewed 240 face-name pairs over two nine-minute fMRI runs. Participants were asked if they thought the name suited the face and responded using a response box. Each run included 120 face-name pairs presented for a duration of 3000 ms, jittered with 34 fixation crosses ranging from 3000–9000 ms in increments of 3000 ms. (b) The retrieval task was then performed on a laptop computer outside the MR scanner. Participants were instructed to choose which of the two names was originally paired with the face shown and then asked if they were confident with their choice. This was used to identify the correct successful encoding trials as remembered (correct) versus lucky guesses.

To evaluate the encoding and retrieval processes of memory, fMRI studies frequently use a recognition memory paradigm that consists of an “encoding” and “recall” phase [5, 68]. Associative memory refers to memory for the relationships between memoranda rather than memory for objects themselves [69–71]. The role of the hippocampus in memory formation has also been specifically linked to associative memory [72]. A specific version of an associative paradigm [17] using face-name pairing known to reliably activate the hippocampus during the successful encoding event and sensitive enough to detect hippocampal dysregulation in a MDD sample [16] was used to investigate activation patterns of the hippocampus during the encoding process inside the MRI scanner (see Figure 1). During the encoding phase inside the MRI, participants were presented with face-name pairs and used a response box provided to indicate if the name suited the face. The retrieval phase was performed after the MRI scan. Participants were presented with a face and two names and instructed to indicate which name was paired with that face during the encoding phase. Participants also rated the confidence of their responses. Trials during the encoding phase were then reclassified based on the responses during the retrieval phase into correct (the participant selected the correct name for the face and indicated a high confidence in their response, suggesting that the face-name association was successfully encoded), guesses (a correct selection with low confidence), or incorrect (the wrong name was selected).

### 2.7. fMRI Scanning Parameters

Participants were scanned on a 3-Tesla Tim trio MRI scanner equipped with a 32-channel phased array head coil. E-prime software version 2.0 (Psychology Software Tools) was used to present stimuli on a rear-projection system (Avotec, Inc., Stuart, FL) in two separate nine-minute functional runs. To obtain optimal hippocampal resolution, all scans were acquired in the oblique coronal plane perpendicular to the long axis of the hippocampus to maximize the anatomic delineation. A total of 416 functional scans were acquired with a *T*_2_^∗^-weighted gradient echo planar imaging sequence (TR =2500 ms, TE = 27 ms, FOV = 192 mm, 3 mm × 3 mm × 3 mm, and flip angle = 70°; in-plane resolution = 3 mm × 3 mm; and 50 slices with 3.5 mm slice thickness). The first 4 volumes of each run were discarded to allow for T1 equilibration. The anatomical scan lasted six minutes and was acquired with a T1 MPRAGE imaging sequence (TR = 2000 ms, TE = 2.63 ms, FOV 256 mm, 1 mm × 1 mm × 1 mm voxels, and flip angle = 9°).

### 2.8. fMRI Analysis

Image preprocessing was performed using SPM12 methods (Statistical Parametric Mapping, Wellcome Department of Cognitive Neurology, London, UK: http://www.fil.ion.ucl.ac.uk/spm) within MatLab 8.3 (The MathWorks Inc., MA). Individual functional images were slice time corrected and realigned to the first image in the series to correct for motion. The EPI images were coregistered to the T1, and segmentation was applied to the T1 anatomical images to extract grey matter, white matter, and CSF masks and calculate a deformation field to transform the data into MNI space. All EPI images were then spatially normalized to the ICBM template using the deformation field, resampled to 3 × 3 × 3, and smoothed using a 6 mm full-width-half maximum isotropic Gaussian filter. General linear model (GLM) was performed at the single-subject level and statistical contrasts were created modeling the hemodynamic response function (HRF) of remembered items with high confidence (correct), remembered items with low confidence (lucky guess), and incorrect trials (incorrect). Six head motion parameters (three rigid body translations and three rotations) were included in the model to reduce the potential effects of motion. Second-level random effects analysis was performed using the contrast of *t*-test of correct > incorrect. Correct retrieval requires well encoding of the items. As such, this contrast which differentiates between poorly and well-encoded trials, known as the subsequent memory effects, is a powerful tool for examining successful memory encoding in the brain [73, 74]. As our primary hypothesis was related to activity in the hippocampus, a hippocampal mask was defined using the automated anatomical labeling (AAL) atlas. Significant clusters from an independent sample *t*-test within the hippocampus (*p* < 0.01 uncorrected, 10 voxels, for this a priori ROI-defining analysis only) for correct > incorrect at baseline were used as an ROI to extract contrast beta values for correct > incorrect in pre- and postscans for each participant. The average beta values from each ROI were imported into SPSS version 20, and a 2 × 2 repeated measures ANOVA (group × time) was run. We then conducted an exploratory whole brain analysis to determine if other brain regions showed task-related effects. For our whole brain analysis, significant clusters were defined as 20 contiguous voxels (180 mm^3^) with *p* < 0.005 uncorrected. A 2 × 2 repeated measures ANOVA (group × time) was run on *β* values for the correct > incorrect contrast in order to identify regions in which there was a difference in pre-post changes across groups. Additionally, an exploratory analysis was run examining a group × time interaction across the whole brain.

## 3. Results

### 3.1. Baseline Characteristics

At baseline, there were no significant differences between groups for BMI, VO_2_max, and MoCA scores (all *p* > 0.05). BDI scores for the MDD group scores indicated severe depression while the healthy group BDI scores indicated no depression (*p* < 0.0001). The depressed group was also older than the healthy group (*p* < 0.0001). See Table 1.

### 3.2. Psychometric, Memory, and Fitness Results

A 2 × 2 repeated measures ANOVA revealed a group × time interaction for BDI scores (*f* (1,15) = 30.42, *p* < 0.0001) indicating that the MDD group had a greater decrease in depression scores pre-post. There were no significant changes in BMI, MoCA scores, or performance on the associative memory task (*p* > 0.05) for either group pre-post. Although baseline memory scores between groups were not significantly different (*p* = 0.477), our results showed that the MDD group performed more poorly on the associative memory task compared to the healthy group (71.48% versus 75.32%) indicating likely memory impairments in the MDD group. One MDD (*n* = 1) participant discontinued baseline VO_2_max testing due to exhaustion and was excluded from VO_2_max analysis. Baseline VO_2_max scores revealed that one MDD participant (*n* = 1) was in the good health benefit rating zone, and the remaining participants (*n* = 14) were in the poor health benefit rating zone based on the Canadian Society for Exercise Physiology guidelines [63]. The healthy group showed a 47% increase in VO_2_max that was significant (*p* = 0.014) while the MDD group showed a marginally significant increase of 31% (*p* = 0.073). There was no significant difference in VO_2_max between groups (*p* = 0.661). These improvements in VO_2_max suggest that the exercise intervention was successful at improving cardiorespiratory fitness (see Table 2).

### 3.3. fMRI Results

**Figure 2:**
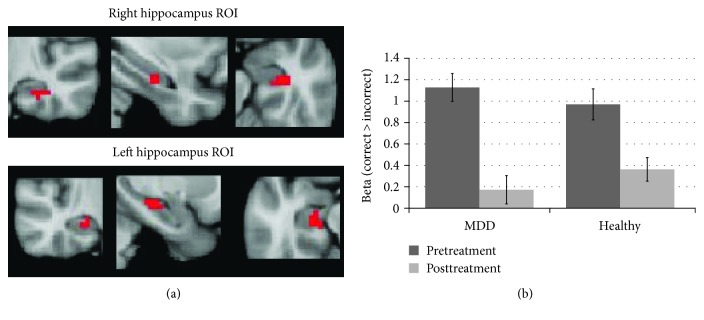
(a) Regions within the hippocampus found to be active in the correct > incorrect contrast for both healthy and MDD at baseline, used as an ROI to extract beta values for an analysis of activity in the hippocampus. (b) Beta values for correct > incorrect from both groups at pretreatment and posttreatment. Both groups showed a reduction in hippocampal activity within the bilateral ROI following exercise. Error bars represent standard error.

Using a main effect contrast of correct > incorrect, collapsing across groups, at baseline, we identified active voxels in the hippocampus and created ROIs for the right and left hippocampus (see Figure 2(a)). A repeated measures ANOVA examining group × time using *β* values for the correct > incorrect contrasts for pre and post revealed a marginal main effect of time (*f* (1,15) = 3.3, *p* = 0.09), no main effect of the group (*f* (1,15) = 0.005, *p* = 0.957) or group × time interaction (*f* (1,15) = 0.165, *p* = 0.69). This marginal main effect of time is driven by a decrease in the correct > incorrect contrast in the hippocampus indicating that there was a decrease in hippocampal activity during successful encoding in both groups following the exercise intervention (see Figure 2(b)).

**Figure 3:**
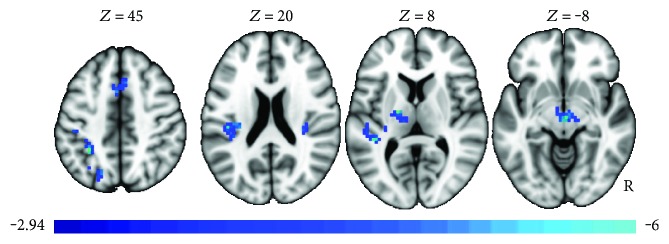
Pre to post changes in neural activity in the correct > incorrect contrast (paired sample *t*-test). Decreases in activity following exercise were noted in several regions, irrespective of group.

The exploratory whole brain analysis did not reveal any clusters in which there were group differences in the pre-post changes following exercise or a main effect of group differences. Given the lack of interaction of the main effects of the group or group × time interaction, a post hoc whole brain analysis was run in which both groups were collapsed. Given the relatively small sample size, this analysis maximizes the power to detect changes in brain activity following the exercise intervention that are common to both healthy and MDD, using the more powerful paired sample *t*-test. Changes in neural activity in the correct > incorrect contrast were compared from the pretreatment to posttreatment MRI scans. Decreases in activity following exercise were noted in several regions (see Figure 3 and Table 3). Regions included a larger cluster in the left posterior insula and smaller clusters in the medial superior frontal/mid cingulate and postcentral superior parietal gyrus.

**Figure 4:**
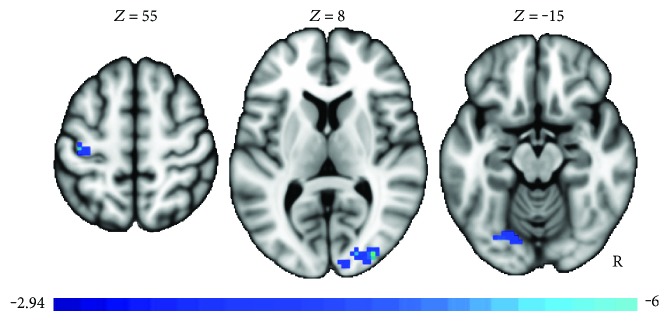
Pre-post changes found a negative relationship between changes in depression scores and activation in the right occipital, left occipital/fusiform, and left precentral gyrus, irrespective of group.

In order to examine regions in which changes in neural activity were related to changes in BDI score, an additional contrast was run, regressing change in BDI against the paired *t*-test described above. Again, in order to maximize power, the healthy and MDD cases were both included in this analysis. The justifications for including both groups are as follows: firstly, although the healthy group was not clinically depressed, there was some pre-post reduction in BDI scores for the healthy group, and secondly, since cases with depression tended to have a larger decrease in BDI, this analysis may be more sensitive to group × time effects, reflected as BDI changes, while also better reflecting areas in which the pre-post differences were behaviorally meaningful. The regression against BDI for pre-post changes found a negative relationship between changes in depression scores and activation in the right occipital, left occipital/fusiform, and left precentral gyrus (see Figure 4 and Table 4).

## 4. Discussion

This small fMRI pilot study used a subsequent memory paradigm to investigate the effects of an eight-week structured, supervised exercise intervention on hippocampal function and overall brain activity in low-active patients with MDD and low-active healthy individuals. The current study yielded two main findings. First, our ROI analysis of the hippocampus showed a marginal decrease in activation for both groups pre-post exercise. Although this decrease in hippocampal activation was only marginally significant, a deactivation pattern was present in both groups and was consistent across other memory-related brain regions noted in the whole brain analysis. These data provide the first evidence that improved cardiovascular fitness, following eight weeks of the minimum recommended dose of exercise, affects neural function alike in healthy and MDD brains. The overall deactivation pattern that we observed in the hippocampus and several other brain regions despite similar memory performance pre-post suggests increased cortical inhibition that attenuated neural activity in a subset of brain regions known to inhibit memory encoding and/or an increase in neural network efficiency during the memory encoding process. Second, our study showed that exercise had a robust antidepressant effect on the MDD group who went from the severe to mild depression range, providing additional support to the growing body of literature that exercise is an effective adjunctive therapy for MDD [75].

A common theme in the neurocognitive literature is that brain activity for remembered items is greater than brain activity for forgotten items, as this suggests successful memory encoding [17, 76–79]. However, neuroimaging studies employing a subsequent memory design have identified a negative relationship between remembered items and neural activity in brain regions such as the insula and the supramarginal gyrus, and hyperactivity in these regions may be detrimental to new memory formation [76, 80–82]. A candidate mechanism for the decrease in neural activity that we observed following the exercise intervention may be a modulation in the main inhibitory neurotransmitter *γ*-aminobutyric acid (GABA). Cortical inhibition, mediated by GABA via cortical interneurons, is an essential mechanism that eliminates task-irrelevant distractors that increase neural noise, which negatively affects attention for task demands. Inhibitory pathways consisting of GABAergic projections between the thalamus and cortex provide a mechanism that may eliminate task-irrelevant distractors by suppressing irrelevant sensory inputs early in sensory processing [83, 84]. A plethora of evidence has identified GABA deficits in MDD, and it has been postulated that GABAergic dysregulation may play a significant role in the pathogenesis of the disorder [85–88]. For example, neuroimaging studies have identified GABA deficits in the dorsolateral prefrontal and occipital cortex in depressed individuals [89–91]. Histopathological studies of postmortem tissue from MDD brains have revealed a reduction in both the density and size of GABAergic neurons in the prefrontal and occipital cortex that conceivably underlie the low levels of GABA seen in neuroimaging studies [92, 93]. Research has shown that exercise may facilitate cortical inhibition by regulating the interplay between glutamatergic excitatory neurons and GABAergic inhibitory interneurons. In mice, running engaged inhibitory mechanisms in the hippocampus through an increased expression of vesicular GABA transporter and extracellular GABA release that was also associated with improved anxiety regulation [94]. In humans with early Parkinson's disease, a neurophysiological study used transcranial magnetic stimulation (TMS) to examine cortical inhibition of the primary motor cortex (M1) following an eight-week, high-intensity aerobics exercise intervention. In addition to improving clinical symptoms, the exercise intervention normalized corticomotor excitability through an increase in GABA-mediated cortical inhibition [95]. Nonetheless, literature supporting the role of exercise in normalizing cortical inhibition via the GABAergic system remains sparse.

Our observed decrease in brain activity during successful memory encoding pre-post the exercise intervention also suggests lowered demands on neural networks and increased neural processing efficiency. Our results provide additional support to a recent body of literature, which postulates that exercise increases neural efficiency. In children, an eight-month aerobics exercise program was associated with decreased activity in several brain regions during an antisaccade task alongside improvements in performance [96]. In elderly adults, a 12-week aerobics exercise program was associated with decreased prefrontal activation despite improvements in visual short-term memory [97]. A similar study conducted in elderly adults with mild cognitive impairment found that 12 weeks of aerobics exercise decreased brain activity in 11 brain regions during memory retrieval despite improvements in memory performance [98]. In adolescence, high-fit individuals showed a pattern of decreased activation in the hippocampus and right superior frontal gyrus combined with a deactivation in the default mode network (DMN) during the encoding of subsequently remembered items, that was absent in low-fit individuals [99]. To determine if aerobics exercise influences learning and memory-associated neural circuitry, a group of researchers examined the brain activity in high-fit and low-fit adolescents during an SME paradigm. Despite comparable memory performance between the two groups, there were notable differences in memory-related and default mode (DMN) brain regions during encoding of successfully remembered word pairs versus forgotten word pairs. Results showed that high-fit individuals displayed a robust deactivation pattern in the DMN areas, such as the ventral medial prefrontal cortex and posterior cingulate cortex, which was absent in the low-fit group. The low-fit group also showed a greater bilateral hippocampal and right superior frontal gyrus activation during encoding of later remembered versus forgotten word pairs. Our results taken together with previous research suggest that improvements in aerobics fitness from the exercise intervention can promote neural processing efficiency during memory encoding processes.

Finally, the neurocognitive benefits associated with exercise may be attributed to increases in cerebral blood flow and neural growth factors, particularly brain-derived neurotrophic factor (BDNF), a key mediator of neuroplasticity in the brain [36, 37]. BDNF, a member of the neurotrophin family, upregulates neurogenesis, promotes neural survival, improves neural structure, and increases synaptic efficacy [100–103]. BDNF also modulates the formation and plasticity of GABAergic synapses and promotes maturation of GABAergic inhibitory networks [104–106]. Reduced BDNF levels are a consistent finding in animal models of depression [107], and administration of exogenous BDNF into the hippocampus is able to produce antidepressant behavioral responses comparable to antidepressant medications [108]. Exercise is known to elevate BDNF production in the hippocampus [35, 109] and has been postulated as a leading candidate mechanism underlying the antidepressant effects of exercise [110, 111].

## 5. Limitations

This pilot study has some limitations. First, the sample size used is rather small and therefore statistically underpowered. We did not age match our groups, which resulted in the MDD group being significantly older than the healthy group. Intrinsically, we wanted to compare MDD brains to young healthy brains with no history of mental health illness or other confounding comorbidities that increase with age and determine if exercise affects neural function in healthy populations who are low active. Also, we did not measure sedentary behavior time which has been shown to have deleterious health consequences independent of daily physical activity levels [112]; as a result, future work must consider sedentary behavior independent of physical activity levels and cardiorespiratory fitness. Another limitation is that our MDD samples were all medicated which may have also affected results. However, this is the typical patient seen in clinical practice, and in any real-world clinical intervention using exercise, the participants would likewise be similarly medicated. Next, even though our analysis collapsing across groups mediates some of the power issues for a transdiagnostic analysis of the effects of exercise on neural activity, the analysis was still underpowered to detect group × time interaction effects.

While we did not closely replicate the results of Fairhall et al., it should be noted that they used a contrast comparing correct trials to fixation, while we made use of a more standard subsequent memory contrast (correct > incorrect). Nevertheless, we did observe a decrease in the correct > incorrect contrast in the hippocampus indicating that there was a decrease in hippocampal activity during successful encoding in both groups following the exercise intervention. We did not however observe any group effects. Given the small sample size, we were likely underpowered to detect any subtle group effects, though it remains possible that the decreases in activity observed following the intervention are common across low-active individuals regardless of diagnostic status. In fact, it should also be noted there was even a small decrease in BDI scores amongst the healthy group. The physiological effects of exercise on the brain may be common amongst both healthy and MDD while still resulting in a substantially greater mood effect for those suffering from depression.

## 6. Future Research

This small pilot study demonstrates that eight weeks of the minimum recommended dose of exercise improved cardiorespiratory and significantly reduced depression severity in the MDD group. Importantly, we were able to demonstrate that combining the minimum recommended dose of exercise with conventional treatments was effective in treating the typical patient seen in the primary care setting who continues to experience severe depressive symptoms despite being treated with antidepressant medication. On the other hand, prescribing exercise to MDD patients presents many challenges to the practitioner since many patients lack motivation to initiate and maintain an exercise routine. Introducing patients to the minimum recommended dose of exercise as an add-on therapy may offer a practical approach for practitioners to help patients initiate and maintain a routine of daily exercise [113, 114].

An interesting finding from this pilot study was that eight weeks of exercise affected healthy and MDD brains similarly. The deactivation pattern we observed in several brain regions warrants further investigation with a larger sample size to allow a more robust statistical analysis. Future work must also include an MDD control group, as this will help us understand the magnitude of the effect of exercise, in combination with other therapies, on depressive symptomology and neural function. Moreover, there is a shortage of reporting sedentary behavior, physical activity levels, and cardiovascular fitness parameters in the MDD literature. As such, some of the differences observed in studies comparing MDD to controls might be confounded by a low-active lifestyle, which may be more prevalent in MDD. To address this gap, future research should compare “fit” and “low-fit” MDD groups to identify markers independent of cardiorespiratory fitness and unique to MDD. Furthermore, the exercise and cognitive literature have not established whether the psychological effects from engaging in exercise, independent of changes in fitness, are still beneficial to mental health and brain function. Future research must indicate whether the effects seen following an exercise intervention are associated with improved cardiovascular fitness or from the psychological benefits from engaging in exercise.

Lastly, MDD is a heterogeneous disorder, and it is likely to be a multifaceted interaction of psychological and neurobiological mechanisms that underlie or mediate the effects of exercise. Future research must consider using a combination approach of multimodal imaging techniques, behavioral assessments, and biochemical analysis to delineate the biological and clinical signatures of fit and unfit MDD populations. Once we are able to elucidate these key biomarkers unique to MDD, novel intervention strategies can then be designed to prevent or reverse neuropsychological pathologies such as MDD.

## Figures and Tables

**Table 1:** Baseline characteristics of participants.

Variables	MDD	*n*	Healthy	*n*	*df*	*p*
Sex (male/female)	1/7	8	4/4	8	1	0.106^a^
Age (years)	37.25 (8.00)	8	20.63 (1.19)	8	14	**<0.0001** ^b^
Body mass index (kg/m^2^)	28.33 (5.12)	8	28.29 (7.91)	8	14	0.993^b^
VO_2_max (ml.kg^−1^.min^−1^)	24.82 (8.00)	7	20.81 (6.48)	8	12	0.326^b^
BDI	41.75 (3.50)	8	5.88 (5.03)	8	14	**<0.0001** ^b^
MoCA	24.63 (1.41)	8	26.13 (3.23)	8	14	0.248^b^

Data are expressed as the mean with the standard deviation in parentheses. ^a^Pearson's chi-square. ^b^Student's *t*-test. VO_2_max: maximum oxygen consumption; BDI: Beck Depression Inventory; MoCA: Montreal Cognitive Assessment.

**Table 2:** Results of pre-post changes for depression scores, cognitive assessment, memory performance, body mass index, and fitness.

Measure	MDD					Healthy					Between-group analysis		
	*n*	Premean (SD)	Postmean (SD)	*p*	*d*	*n*	Premean (SD)	Postmean (SD)	*p*	*d*	F	*p*	*d*_ppc2_
BDI	8	41.75 (3.50)	15.50 (10.43)	**0.0004**	2.89	8	5.88 (5.03)	3.25 (4.53)	0.072	0.545	30.42	**<0.0001**	5.34
MOCA	8	24.63 (1.41)	25.75 (2.38)	0.229	0.564	8	26.13 (3.23)	27.13 (1.81)	0.286	0.338	0.011	0.920	0.047
Memory task (high confidence (correct) %)	8	71.48 (9.50)	69.08 (12.03)	0.359	0.210	8	75.32 (9.29)	75.13 (9.07)	0.942	0.020	0.418	0.529	0.230
BMI (kg/m^2^)	8	28.33 (5.12)	28.29 (4.48)	0.934	0.007	8	28.29 (7.91)	27.95 (6.61)	0.530	0.023	0.205	0.657	0.044
VO_2_max (ml.kg^−1^.min^−1^)	7	24.82 (8.00)	32.52 (10.12)	0.073	0.834	8	20.81 (6.48)	30.57 (8.66)	**0.014**	1.26	7.96	0.661	0.279

Data are expressed as mean with SD in parentheses. BDI: Beck Depression Inventory; MoCA: Montreal Cognitive Assessment; BMI: body mass index; VO_2_max: maximum oxygen consumption; *d*: Cohen's *d*.

**Table 3:** Brain regions showing pre-post changes in activity for the correct > incorrect, irrespective of group.

Voxels	Peak *T*	MNI coordinates			BA	Location
		*X*	*Y*	*Z*		
37	−6.27	−6	17	41	32	Medial superior frontal/mid cingulate
35	−5.85	−18	−10	8		Left putamen
35	−5.79	−30	−46	44	40	Left supramarginal/intraparietal sulcus
45	−5.61	−42	−28	35	3	Postcentral, superior parietal gyrus
50	−5.34	3	−16	−7		Thalamus/midbrain
139	−5.3	−42	−37	8	41	Left posterior insula
30	−5.22	−18	−70	47	7	Left superior parietal
36	−5.17	30	−28	14		Right posterior insula
20	−4.75	54	−43	−4	21	Right posterior mid temporal
25	−3.82	12	−82	−7	18	Right occipital gyrus

MNI: Montreal Neurological Institute; BA: Broadmann area.

**Table 4:** Regions showing decreased activation associated with depression, irrespective of group.

Voxels	Peak *T*	MNI coordinates			BA	Location
		*X*	*Y*	*Z*		
58	−6.41	36	−88	11	19	Right occipital
27	−4.92	−39	−22	56	4	Left precentral gyrus
29	−4.1	−24	−73	−13	18	Left occipital/fusiform

MNI: Montreal Neurological Institute; BA: Broadmann area.

## References

[1] Airaksinen E., Larsson M., Lundberg I., Forsell Y. (2004). Cognitive functions in depressive disorders: evidence from a population-based study. *Psychological Medicine*.

[2] Shilyansky C., Williams L. M., Gyurak A., Harris A., Usherwood T., Etkin A. (2016). Effect of antidepressant treatment on cognitive impairments associated with depression: a randomised longitudinal study. *The Lancet Psychiatry*.

[3] Ilsley J. E., Moffoot A. P. R., O’Carroll R. E. (1995). An analysis of memory dysfunction in major depression. *Journal of the Affective Disorders*.

[4] Sweeney J., Kmeic J., Kupfer D. (2000). Neuropsychologic impairments in bipolar and unipolar mood disorders on the CANTAB neurocognitive battery. *Biological Psychiatry*.

[5] Sperling R., Chua E., Cocchiarella A. (2003). Putting names to faces: successful encoding of associative memories activates the anterior hippocampal formation. *NeuroImage*.

[6] Zakzanis K. K., Leach L., Kaplan E. (1998). On the nature and pattern of neurocognitive function in major depressive disorder. *Neuropsychiatry, Neuropsychology, & Behavioral Neurology*.

[7] Tulving E., Markowitsch H. J. (1998). Episodic and declarative memory: role of the hippocampus. *Hippocampus*.

[8] Burt D., Zembar M., Niederehe G. (1995). Depression and memory impairment: a meta-analysis of the association, its pattern, and specificity. *Psychological Bulletin*.

[9] Heckers S., Weiss A. P., Alpert N. M., Schacter D. L. (2002). Hippocampal and brain stem activation during word retrieval after repeated and semantic encoding. *Cerebral Cortex*.

[10] Bray N. (2014). Learning and memory: the hippocampus encodes objects in time. *Nature Reviews Neuroscience*.

[11] Köhler S., Moscovitch M., Winocur G., McIntosh A. R. (2000). Episodic encoding and recognition of pictures and words: role of the human medial temporal lobes. *Acta Psychologica*.

[12] Zola S. M., Squire L. R., Teng E., Stefanacci L., Buffalo E. A., Clark R. E. (2000). Impaired recognition memory in monkeys after damage limited to the hippocampal region. *Journal of Neuroscience*.

[13] Scovile W. B., Milner B. (1957). Loss of recent memory after bilateral hippocampal lesions. *Journal of Neurology, Neurosurgery, and Psychiatry*.

[14] Videbech P., Ravnkilde B. (2004). Hippocampal volume and depression: a meta-analysis of MRI studies. *American Journal of Psychiatry*.

[15] McKinnon M. C., Yucel K., Nazarov A., MacQueen G. M. (2009). A meta-analysis examining clinical predictors of hippocampal volume in patients with major depressive disorder. *Journal of Psychiatry & Neuroscience: JPN*.

[16] Schmaal L., Veltman D. J., van Erp T. G. (2016). Subcortical brain alterations in major depressive disorder: findings from the ENIGMA major depressive disorder working group. *Molecular Psychiatry*.

[17] Fairhall S. L., Sharma S., Magnusson J., Murphy B. (2010). Memory related dysregulation of hippocampal function in major depressive disorder. *Biological Psychology*.

[18] Milne A. M. B., MacQueen G. M., Hall G. B. C. (2012). Abnormal hippocampal activation in patients with extensive history of major depression: an fMRI study. *Journal of Psychiatry & Neuroscience: JPN*.

[19] Toki S., Okamoto Y., Onoda K. (2014). Hippocampal activation during associative encoding of word pairs and its relation to symptomatic improvement in depression: a functional and volumetric MRI study. *Journal of Affective Disorders*.

[20] Rajkowska G., Miguel-Hidalgo J. J. (2007). Gliogenesis and glial pathology in depression. *CNS & Neurological Disorders Drug Targets*.

[21] Harrison P. J. (2002). The neuropathology of primary mood disorder. *Brain*.

[22] Pittenger C., Duman R. S. (2007). Stress, depression, and neuroplasticity: a convergence of mechanisms. *Neuropsychopharmacology*.

[23] Stockmeier C. A., Mahajan G. J., Konick L. C. (2004). Cellular changes in the postmortem hippocampus in major depression. *Biological Psychiatry*.

[24] Qiao H., An S.-C., Ren W., Ma X.-M. (2014). Progressive alterations of hippocampal CA3-CA1 synapses in an animal model of depression. *Behavioural Brain Research*.

[25] Andrews G., Sanderson K., Corry J., Lapsley H. M. (2000). Using epidemiological data to model efficiency in reducing the burden of depression. *The Journal of Mental Health Policy and Economics*.

[26] Fava G. A., Ruini C. (2002). Long-term treatment of depression: there is more than drugs. *Recenti Progressi in Medicina*.

[27] Diverty B., Beaudet M. P. (1997). Depression: an undertreated disorder. *Health Reports*.

[28] Insel T. R., Charney D. S. (2003). Research on major depression: strategies and priorities. *The Journal of the American Medical Association*.

[29] Babyak M., Blumenthal J. A., Herman S. (2000). Exercise treatment for major depression: maintenance of therapeutic benefit at 10 months. *Psychosomatic Medicine*.

[30] Blumenthal J. A., Babyak M. A., Moore K. A. (1999). Effects of exercise training on older patients with major depression. *Archives of Internal Medicine*.

[31] Blumenthal J. A., Babyak M. A., Doraiswamy P. M. (2007). Exercise and pharmacotherapy in the treatment of major depressive disorder. *Psychosomatic Medicine*.

[32] Martinsen E. W., Medhus A., Sandvik L. (1985). Effects of aerobic exercise on depression: a controlled study. *British Medical Journal*.

[33] Stathopoulou G., Powers M. B., Berry A. C., Smits J. A. J., Otto M. W. (2006). Exercise interventions for mental health: a quantitative and qualitative review. *Clinical Psychology: Science and Practice*.

[34] Schuch F. B., Vancampfort D., Richards J., Rosenbaum S., Ward P. B., Stubbs B. (2016). Exercise as a treatment for depression: a meta-analysis adjusting for publication bias. *Journal of Psychiatric Research*.

[35] Cotman C. W., Berchtold N. C. (2002). Exercise: a behavioral intervention to enhance brain health and plasticity. *Trends in Neurosciences*.

[36] van Praag H., Kempermann G., Gage F. H. (1999). Running increases cell proliferation and neurogenesis in the adult mouse dentate gyrus. *Nature Neuroscience*.

[37] Vivar C., Potter M. C., van Praag H. (2013). All about running: synaptic plasticity, growth factors and adult hippocampal neurogenesis. *Current Topics in Behavioral Neurosciences*.

[38] van Praag H., Shubert T., Zhao C., Gage F. H. (2005). Exercise enhances learning and hippocampal neurogenesis in aged mice. *Journal of Neuroscience*.

[39] van Praag H. (2008). Neurogenesis and exercise: past and future directions. *Neuromolecular Medicine*.

[40] Kobilo T., Liu Q.-R., Gandhi K., Mughal M., Shaham Y., van Praag H. (2011). Running is the neurogenic and neurotrophic stimulus in environmental enrichment. *Learning & Memory (Cold Spring Harbor, N.Y.)*.

[41] Dietrich M. O., Andrews Z. B., Horvath T. L. (2008). Exercise-induced synaptogenesis in the hippocampus is dependent on UCP2-regulated mitochondrial adaptation. *Journal of Neuroscience*.

[42] Lopez-Lopez C., LeRoith D., Torres-Aleman I. (2004). Insulin-like growth factor I is required for vessel remodeling in the adult brain. *Proceedings of the National Academy of Sciences of the United States of America*.

[43] Redila V. A., Christie A. B. R. (2006). Exercise-induced changes in dendritic structure and complexity in the adult hippocampal dentate gyrus. *Neuroscience*.

[44] Yau S.-Y., Lau B. W., Tong J. B. (2011). Hippocampal neurogenesis and dendritic plasticity support running-improved spatial learning and depression-like behaviour in stressed rats. *PLoS One*.

[45] Erickson K. I., Voss M. W., Prakash R. S. (2011). Exercise training increases size of hippocampus and improves memory. *Proceedings of the National Academy of Sciences of the United States of America*.

[46] Anderson-Hanley C., Arciero P. J., Brickman A. M. (2012). Exergaming and older adult cognition: a cluster randomized clinical trial. *American Journal of Preventive Medicine*.

[47] Voss M. W., Prakash R. S., Erickson K. I. (2010). Plasticity of brain networks in a randomized intervention trial of exercise training in older adults. *Frontiers in Aging Neuroscience*.

[48] Voss M. W., Heo S., Prakash R. S. (2013). The influence of aerobic fitness on cerebral white matter integrity and cognitive function in older adults: results of a one-year exercise intervention. *Human Brain Mapping*.

[49] Young J., Angevaren M., Rusted J., Tabet N., Young J. (2015). Aerobic exercise to improve cognitive function in older people without known cognitive impairment. *Cochrane Database of Systematic Reviews*.

[50] Gates N., Fiatarone Singh M. A., Sachdev P. S., Valenzuela M. (2013). The effect of exercise training on cognitive function in older adults with mild cognitive impairment: a meta-analysis of randomized controlled trials. *The American Journal of Geriatric Psychiatry*.

[51] Smith P. J., Blumenthal J. A., Hoffman B. M. (2010). Aerobic exercise and neurocognitive performance: a meta-analytic review of randomized controlled trials. *Psychosomatic Medicine*.

[52] Sink K. M., Espeland M. A., Castro C. M. (2015). Effect of a 24-month physical activity intervention vs health education on cognitive outcomes in sedentary older adults. *The Journal of the American Medical Association*.

[53] Colcombe S. J., Erickson K. I., Scalf P. E. (2006). Aerobic exercise training increases brain volume in aging humans. *The Journals of Gerontology Series A: Biological Sciences and Medical Sciences*.

[54] Erickson K. I., Prakash R. S., Voss M. W. (2009). Aerobic fitness is associated with hippocampal volume in elderly humans. *Hippocampus*.

[55] Pajonk F. G., Wobrock T., Gruber O. (2010). Hippocampal plasticity in response to exercise in schizophrenia. *Archives of General Psychiatry*.

[56] American Psychiatric Association, and American Psychiatric Association (2000). *Task Force on DSM-IV, Diagnostic and Statistical Manual of Mental Disorders: DSM-IV-TR*.

[57] Beck A., Steer R., Brown G. (1996). *BDI-II. Beck Depression Inventory Second Edition. Manual*.

[58] Nasreddine Z. S., Phillips N. A., Bédirian V. (2005). The Montreal Cognitive Assessment, MoCA: a brief screening tool for mild cognitive impairment. *Journal of the American Geriatrics Society*.

[59] Beck A. T., Steer R. A., Ball R., Ranieri W. F. (1996). Comparison of Beck depression inventories-IA and-II in psychiatric outpatients. *Journal of Personality Assessment*.

[60] Beekley M., Brechue W., Dehoyos D. (2004). Cross-validation of the YMCA submaximal cycle ergometer test to predict VO2max. *Research Quarterly for Exercise and Sport*.

[61] Golding L. A., Myers C. R., Sinning W. E. (1989). *Y’s way to Physical Fitness: The Complete Guide to Fitness Testing and Instruction*.

[62] Pescatello L. S., American College of Sports Medicine (2014). *ACSM’s Guidelines for Exercise Testing and Prescription*.

[63] Taylor A. W. (2004). Canada’s physical activity guide to healthy active living for older adults. *Journal of Aging and Physical Activity*.

[64] Garber C. E., Blissmer B., Deschenes M. R. (2011). American College of Sports Medicine position stand. Quantity and quality of exercise for developing and maintaining cardiorespiratory, musculoskeletal, and neuromotor fitness in apparently healthy adults: guidance for prescribing exercise. *Medicine and Science in Sports and Exercise*.

[65] Stanton R., Reaburn P. (2014). Exercise and the treatment of depression: a review of the exercise program variables. *Journal of Science and Medicine in Sport*.

[66] Cooney G. (2013). Exercise for depression. *Journal of Evidence-Based Medicine*.

[67] Morris S. B. (2008). Estimating effect sizes from the pretest-posttest-control group designs. *Organizational Research Methods*.

[68] Henson R. (2005). A mini-review of fMRI studies of human medial temporal lobe activity associated with recognition memory. *Quarterly Journal of Experimental Psychology B*.

[69] Murray L. J., Ranganath C. (2007). The dorsolateral prefrontal cortex contributes to successful relational memory encoding. *Journal of Neuroscience*.

[70] Blumenfeld R. S., Ranganath C. (2006). Dorsolateral prefrontal cortex promotes long-term memory formation through its role in working memory organization. *Journal of Neuroscience*.

[71] Achim A. M., Lepage M. (2005). Neural correlates of memory for items and for associations: an event-related functional magnetic resonance imaging study. *Journal of Cognitive Neuroscience*.

[72] Konkel A., Cohen N. J. (2009). Relational memory and the hippocampus: representations and methods. *Frontiers in Neuroscience*.

[73] Paller K. A., Wagner A. D. (2002). Observing the transformation of experience into memory. *Trends in Cognitive Sciences*.

[74] Wagner A. D., Schacter D. L., Rotte M. (1998). Building memories: remembering and forgetting of verbal experiences as predicted by brain activity. *Science*.

[75] Mura G., Moro M. F., Patten S. B., Carta M. G. (2014). Exercise as an add-on strategy for the treatment of major depressive disorder: a systematic review. *CNS Spectrums*.

[76] Wagner A. D., Davachi L. (2001). Cognitive neuroscience: forgetting of things past. *Current Biology*.

[77] Wagner A. D., Davachi L. (2001). Cognitive neuroscience: forgetting of things past. *Current Biology*.

[78] Morcom A. M., Good C. D., Frackowiak R. S. J., Rugg M. D. (2003). Age effects on the neural correlates of successful memory encoding. *Brain*.

[79] Kirchhoff B. A., Wagner A. D., Maril A., Stern C. E. (2000). Prefrontal-temporal circuitry for episodic encoding and subsequent memory. *Journal of Neuroscience*.

[80] Otten L. J., Rugg M. D. (2001). When more means less: neural activity related to unsuccessful memory encoding. *Current Biology*.

[81] Daselaar S. M., Prince S. E., Cabeza R. (2004). When less means more: deactivations during encoding that predict subsequent memory. *NeuroImage*.

[82] Cabeza R., Grady C. L., Nyberg L. (1997). Age-related differences in neural activity during memory encoding and retrieval: a positron emission tomography study. *The Journal of Neuroscience*.

[83] Knight R. T., Grabowecky M. F., Scabini D. (1995). Role of human prefrontal cortex in attention control. *Advances in Neurology*.

[84] Guillery R. W., Feig S. L., Lozsádi D. A. (1998). Paying attention to the thalamic reticular nucleus. *Trends in Neurosciences*.

[85] Sanacora G., Saricicek A. (2007). GABAergic contributions to the pathophysiology of depression and the mechanism of antidepressant action. *CNS & Neurological Disorders-Drug Targets*.

[86] Luscher B., Shen Q., Sahir N. (2011). The GABAergic deficit hypothesis of major depressive disorder. *Molecular Psychiatry*.

[87] Cryan J. F., Kaupmann K. (2005). Don’t worry ‘B’ happy!: a role for GABAB receptors in anxiety and depression. *Trends in Pharmacological Sciences*.

[88] Bajbouj M., Lisanby S. H., Lang U. E., Danker-Hopfe H., Heuser I., Neu P. (2006). Evidence for impaired cortical inhibition in patients with unipolar major depression. *Biological Psychiatry*.

[89] Bhagwagar Z., Wylezinska M., Jezzard P. (2007). Reduction in occipital cortex γ-aminobutyric acid concentrations in medication-free recovered unipolar depressed and bipolar subjects. *Biological Psychiatry*.

[90] Sanacora G., Mason G. F., Rothman D. L. (2003). Increased cortical GABA concentrations in depressed patients receiving ECT. *The American Journal of Psychiatry*.

[91] Hasler G., van der Veen J. W., Tumonis T., Meyers N., Shen J., Drevets W. C. (2007). Reduced prefrontal glutamate/glutamine and γ-aminobutyric acid levels in major depression determined using proton magnetic resonance spectroscopy. *Archives of General Psychiatry*.

[92] Maciag D., Hughes J., O'Dwyer G. (2010). Reduced density of calbindin immunoreactive GABAergic neurons in the occipital cortex in major depression: relevance to neuroimaging studies. *Biological Psychiatry*.

[93] Rajkowska G., O’Dwyer G., Teleki Z., Stockmeier C. A., Miguel-Hidalgo J. J. (2007). GABAergic neurons immunoreactive for calcium binding proteins are reduced in the prefrontal cortex in major depression. *Neuropsychopharmacology*.

[94] Schoenfeld T. J., Rada P., Pieruzzini P. R., Hsueh B., Gould E. (2013). Physical exercise prevents stress-induced activation of granule neurons and enhances local inhibitory mechanisms in the dentate gyrus. *The Journal of Neuroscience*.

[95] Fisher B. E., Wu A. D., Salem G. J. (2008). The effect of exercise training in improving motor performance and corticomotor excitability in people with early Parkinson’s disease. *Archives of Physical Medicine and Rehabilitation*.

[96] Krafft C. E., Schwarz N. F., Chi L. (2014). An 8-month randomized controlled exercise trial alters brain activation during cognitive tasks in overweight children. *Obesity*.

[97] Nishiguchi S., Yamada M., Tanigawa T. (2015). A 12-week physical and cognitive exercise program can improve cognitive function and neural efficiency in community-dwelling older adults: a randomized controlled trial. *Journal of the American Geriatrics Society*.

[98] Smith J. C., Nielson K. A., Antuono P. (2013). Semantic memory functional MRI and cognitive function after exercise intervention in mild cognitive impairment. *Journal of Alzheimer’s Disease*.

[99] Herting M. M., Nagel B. J. (2013). Differences in brain activity during a verbal associative memory encoding task in high- and low-fit adolescents. *Journal of Cognitive Neuroscience*.

[100] Knaepen K., Goekint M., Heyman E. M., Meeusen R. (2010). Neuroplasticity—exercise-induced response of peripheral brain-derived neurotrophic factor. *Sports Medicine*.

[101] Scharfman H., Goodman J., Macleod A., Phani S., Antonelli C., Croll S. (2005). Increased neurogenesis and the ectopic granule cells after intrahippocampal BDNF infusion in adult rats. *Experimental Neurology*.

[102] Kubo T., Nonomura T., Enokido Y., Hatanaka H. (1995). Brain-derived neurotrophic factor (bdnf) can prevent apoptosis of rat cerebellar granule neurons in culture. *Developmental Brain Research*.

[103] Kellner Y., Gödecke N., Dierkes T., Thieme N., Zagrebelsky M., Korte M. (2014). The BDNF effects on dendritic spines of mature hippocampal neurons depend on neuronal activity. *Frontiers in Synaptic Neuroscience*.

[104] Gottmann K., Mittmann T., Lessmann V. (2009). BDNF signaling in the formation, maturation and plasticity of glutamatergic and GABAergic synapses. *Experimental Brain Research*.

[105] Waterhouse E. G., An J. J., Orefice L. L. (2012). BDNF promotes differentiation and maturation of adult-born neurons through GABAergic transmission. *The Journal of Neuroscience*.

[106] Hong E. J., McCord A. E., Greenberg M. E. (2008). A biological function for the neuronal activity-dependent component of Bdnf transcription in the development of cortical inhibition. *Neuron*.

[107] Song L., Che W., Min-wei W., Murakami Y., Matsumoto K. (2006). Impairment of the spatial learning and memory induced by learned helplessness and chronic mild stress. *Pharmacology Biochemistry and Behavior*.

[108] Shirayama Y., Chen A. C. H., Nakagawa S., Russell D. S., Duman R. S. (2002). Brain-derived neurotrophic factor produces antidepressant effects in behavioral models of depression. *Journal of Neuroscience*.

[109] Oliff H. S., Berchtold N. C., Isackson P., Cotman C. W. (1998). Exercise-induced regulation of brain-derived neurotrophic factor (BDNF) transcripts in the rat hippocampus. *Molecular Brain Research*.

[110] Cotman C. W., Berchtold N. C., Christie L. (2007). Exercise builds brain health: key roles of growth factor cascades and inflammation. *Trends in Neurosciences*.

[111] Duman C. H., Schlesinger L., Russell D. S., Duman R. S. (2008). Voluntary exercise produces antidepressant and anxiolytic behavioral effects in mice. *Brain Research*.

[112] Hamilton M. T., Healy G. N., Dunstan D. W., Zderic T. W., Owen N. (2008). Too little exercise and too much sitting: inactivity physiology and the need for new recommendations on sedentary behavior. *Current Cardiovascular Risk Reports*.

[113] DiMatteo M. R., Lepper H. S., Croghan T. W. (2000). Depression is a risk factor for noncompliance with medical treatment: meta-analysis of the effects of anxiety and depression on patient adherence. *Archives of Internal Medicine*.

[114] Blumenthal J. A., Smith P. J., Hoffman B. M. (2012). Opinion and evidence: is exercise a viable treatment for depression?. *ACSM’s Health and Fitness Journal*.

